# The new Rapid-i carrier is an effective system for human embryo vitrification at both the blastocyst and cleavage stage

**DOI:** 10.1186/1477-7827-11-41

**Published:** 2013-05-15

**Authors:** Nina N Desai, Jeffrey M Goldberg, Cynthia Austin, Tommaso Falcone

**Affiliations:** 1 Department of OB/GYN/Women’s Health Institute, Cleveland Clinic Fertility Center, Beachwood, OH, USA

## Abstract

**Background:**

The Rapid-i is a new FDA cleared closed carrier for embryo vitrification. The cooling rate of - 1220°C/min is far lower than that reported with open vitrification systems such as the cryoloop (−15,000°C/min). Little published data is currently available on this device. This study presents our initial clinical data, as well as live birth outcomes, with the Rapid-i. The efficacy of this device for the cryopreservation of cleavage, as well as blastocyst stage human embryos is also analyzed. We further compare outcomes to those achieved with the cryoloop, an “open” vitrification system routinely used in our laboratory.

**Methods:**

Human embryos were vitrified at either the 8–10 cell stage or else the blastocyst stage. The vitrification protocol was: 7.5% DMSO/7.5% ethylene glycol (EG) (2–3 min) followed by incubation in 15% DMSO /15% EG (45 sec) before loading on the vitrification carrier. Cryoprotectant was removed during warming by sequential washes in 0.25 M and 0.125 M sucrose in culture medium. Clinical outcome data for frozen cycles between January 2011 and August 2012 were stratified according to carrier and cell stage. The student *t*-test and chi square test were used to compare results. P value of < 0.05 was considered significant.

**Results:**

A total of 486 vitrified-warmed embryos were assessed and 92% of them were transferred. The clinical pregnancy rate (CPR) and implantation rate (IR) with Rapid-i vitrified blastocysts were 59% and 49%, versus 47% and 37%, respectively for cleavage stage embryos. This was not statistically different from results with the cryoloop vitrified blastocysts (CPR 46%, IR 38%) nor the cleavage stage vitrified embryos (CPR 49%, IR 35%). To date, there have been 31 deliveries of 34 healthy infants from Rapid-i vitrified embryos, with another 12 pregnancies still on-going.

**Conclusions:**

The Rapid-i offers an excellent alternative to existing open vitrification devices for embryo cryopreservation at the 8–10 cell stage as well as the blastocyst stage. Use of this type of “closed” sealed system that prevents direct contact between the embryos and liquid nitrogen reduces the potential risk of sample cross-contamination or infection. These preliminary data and live birth outcomes have paved the way toward transitioning to a closed vitrification system in our own IVF program.

## Background

Vitrification is rapidly replacing slow cryopreservation as the method of choice for embryo freezing in clinics world-wide. Increasing evidence from published outcomes with both techniques suggest that vitrification may in fact be superior to slow cooling [1-7].

In contrast to slow cryopreservation methods, which use cooling rates of - 0.3°C per minute, vitrification uses rapid cooling rates to preserve the embryo instantaneously in a “glass-like state”. Vitrification requires high concentrations of cryoprotectant combined with high cooling rates to transition from a fluid to solid state without a phase change thus avoiding intra or extracellular ice crystallization [8]. A practical limitation to establishing successful vitrification protocols has been the achievement of sufficiently high cooling rates in conventional freezing vessels [7,9,10]. Developing vessels to sequester the cell in miniscule fluid volumes, of a microliter or less, has been one approach to increasing cooling rates.

Human embryos have been cryopreserved with a variety of different vitrification devices or carriers that can be classified as either “open” or “closed”. “Open” vitrification devices allow direct contact of the embryos with liquid nitrogen. Much of the early work establishing the efficacy of vitrification, and documenting the first live births, were performed using open carriers like the cryoloop [11-13], EM grids [14], Cryotop [15] and open pulled straws [16-19]. Despite successful use of “open” carriers for embryo vitrification, direct contact between the embryo and liquid nitrogen may not be desirable as there is a potential risk of cross-contamination between specimens or inadvertent exposure to contaminants present in the tank [20-22]. The use of “closed” vitrification carriers circumvents these risks. Examples of closed carrier systems include; cryotips (Irvine Scientific, CA, USA), high security vitrification straw (HSV) (Cryo BioSystem, Paris, France), VitriSafe (VitriMed, Austria) and Cryopette (Origio, Denmark). Initial concerns that the lower cooling rates with closed sealed vitrification systems may compromise pregnancy outcomes have been dispelled by recent publications using commercially marketed “closed” vitrification carriers [2,23-26].

The Rapid-i is a new “closed“ vitrification device, recently received FDA clearance for embryo vitrification. The cooling rate of −1220°C/min is far lower than that reported with other vitrification systems [26,27]. Little published data is currently available on this new carrier [28-30]. The present work summarizes our initial clinical data, as well as live birth outcomes, with the Rapid-i. The efficacy of this device for the cryopreservation of early, as well as late stage, human embryos is also analyzed. We further compare outcomes to those achieved in our laboratory with an “open” vitrification system, namely the cryoloop, with its high cooling rate of −15,000°C /min.

## Methods

### Patients

Embryo vitrification using the Rapid- i carrier was introduced into our clinical practice in January 2011 for cryopreservation of day 3 cleavage stage embryos as well as blastocysts. Prior to this, embryo vitrification was performed using the cryoloop.

This study examines outcomes from patients ≤40 years of age returning to the Cleveland Clinic Fertility Center for a frozen embryo transfer (FET) cycle between January 2011 and August 2012. During this time period, 95 couples returned for an FET cycle with embryos vitrified with the new Rapid-i carrier. Another 161 couples had an FET cycle with embryos vitrified prior to 2011 using the cryoloop carrier. Outcome data was retrospectively analyzed from our IVF laboratory database registry in accordance with the ethical policy and guidelines set forth by the Cleveland Clinic’s Institutional Review Board.

### Ovarian stimulation and IVF

Women were treated with either a GnRH-agonist (gonadotropin releasing hormone) or a GnRH-antagonist to suppress ovulation until follicle maturity was attained. Ovarian stimulation was initiated using daily injections of follicle stimulating hormone (FSH) at starting doses of 225 IU per day or adjusted based on serum anti-mullerian hormone levels, antral follicle counts and the response to previous stimulations. Subsequent doses were adjusted according to transvaginal ultrasonograms and serum estradiol levels. Final follicular maturation was triggered with human chorionic gonadotrophin (hCG) when at least two lead follicles measured 18 mm in mean diameter. Oocytes were collected 36 hours later by transvaginal needle aspiration of follicles under ultrasound guidance.

Mature oocytes were inseminated by intracytoplasmic sperm injection 2–4 hours after retrieval. Intermediate and immature oocytes underwent conventional IVF insemination. Oocytes were examined 16–18 hours after insemination for the presence of two pronuclei. Zygotes were individually cultured in 20 μl drops of Global medium (LifeGlobal, Guilford, CT) supplemented with 10% Synthetic Protein Supplement (SPS; Cooper Surgical; Trumbull, CT) under an oil overlay. All culture was performed at 37°C with 6% CO_2_ in air. Fresh embryo transfers were performed on day 5 for patients with 8 or more good quality embryos and on day 3 for all other patients.

### Embryo assessment

Embryos were observed and graded daily. Cleavage stage embryos were assessed for cell stage, percent fragmentation, multinucleation, blastomere symmetry and degree of cell:cell adherence. Blastocyst grade was assigned based on day of blastocyst formation, blastocyst maturity, inner cell mass development, trophectoderm appearance and degree of necrosis after warming using a previously described scoring system [31,32]. Blastocoel volume and expansion were used to classify blastocysts as: A = early blastocyst, cavity just starting to form; B = early blastocyst, cavity less than half the volume of embryo; C = expanded with cavity greater than half embryo volume; D = fully expanded; and E = hatching. The inner cell mass was graded as: 0- absent or not yet visible; 1- sparse, few cells; and 2- well defined, discrete cell mass. Trophectoderm of the blastocyst was assessed based on overall cell number and organization: 1- low cell number, stretched appearance; 2- well organized cohesive cell layer; and 3- extremely high cell number but well organized.

Embryo cryopreservation was carried out on day 3 at the cleavage stage or on days 5/6 at the blastocyst stage. For day 3 cryopreservation, embryos needed to be between 8–10 cells with even blastomeres and <20% fragmentation. Embryos not meeting these criteria were kept in extended culture and vitrified if they reached the blastocyst stage. Patients having a fresh blastocyst transfer had their supernumerary blastocysts (grades B-E), which displayed an inner cell mass, cryopreserved on day 5. The remaining embryos were given an extra day to develop in culture. In a few patients, morula and early blastocysts without a clearly visible ICM were cryopreserved if nothing else was available for cryobanking by day 6.

### Vitrification procedure

A two-step vitrification protocol was used for both cleavage and blastocyst stage embryos [33]. This technique uses dimethylsulfoxide (DMSO), ethylene glycol (EG) and sucrose as the cryoprotectant agents (CPA). Embryos were loaded either singly or in pairs on to the carrier and vitrified. All vitrification solutions were prepared in-house. Vitrification Solution #1 (VS1) consisted of 7.5% DMSO and 7.5% EG in Global medium with 20% SPS. Vitrification Solution #2 (VS2) contained 15% DMSO, 15% EG, 10 mg/ml Ficoll-70 and 0.65 M sucrose. All incubations steps were performed at 37°C. Embryos were rinsed and held in VS1 for 2 min (3 min for blastocysts). Embryos were then quickly moved through three drops of VS2, holding for 15 sec in each drop, before loading on the vitrification carrier in use at the time.

**Figure 1:**
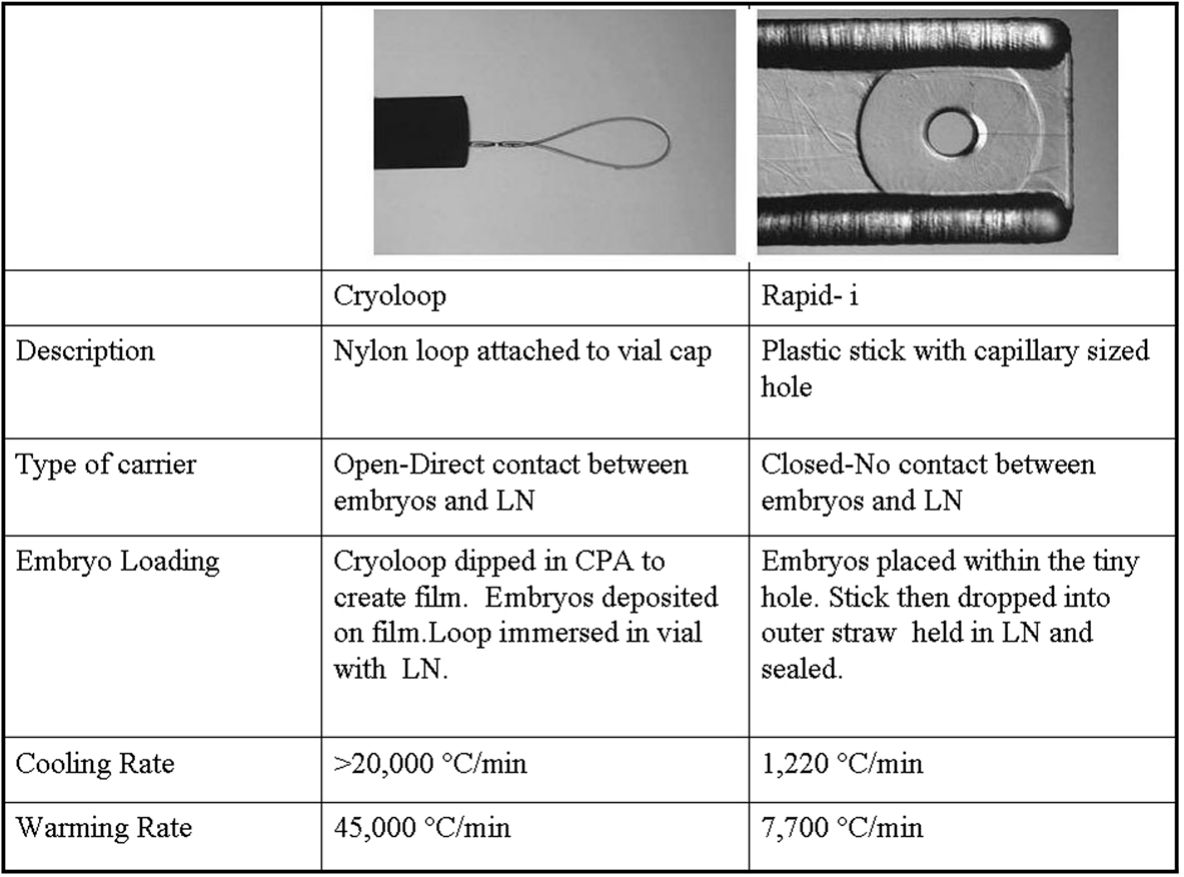
**Comparison of the “closed” Rapid-i carrier to the “open” cryoloop.** Diagram illustrates the two carriers, properties and compares cooling /warming rates.

The Rapid-i was prepared for loading in accordance with the manufacturer’s instructions. The outer straw with metal rod was immersed in the liquid nitrogen holding container. The Rapid-i stick with hole was laid on the lid of a Falcon 1006 dish. A finely drawn glass micropipette was used to pick up 1–2 embryos. The tiny hole in the Rapid-i stick was visualized using a dissecting microscope and the embryos were deposited with minimal fluid into the hole (Figure 1). The Rapid-i stick with embryos was quickly dropped into the pre-frozen outer straw after first removing the metal rod. The outer straw containing the Rapid-i stick was then quickly sealed with an ultrasonic sealer. Straws were placed in goblets then moved to the liquid nitrogen storage tank.

The technique for cryoloop loading involved first preparing a film of cryoprotectant on the loop by dipping in VS2. The embryos were then quickly loaded on to the film using a micropipette (Figure 1). Immediately after loading, the cryoloop with embryos was immersed in a cryovial containing liquid nitrogen. The magnetic cap was tightened using a metal wand and the vial was then moved to the liquid nitrogen storage tank.

### Artificial collapse of blastocysts

**Figure 2:**
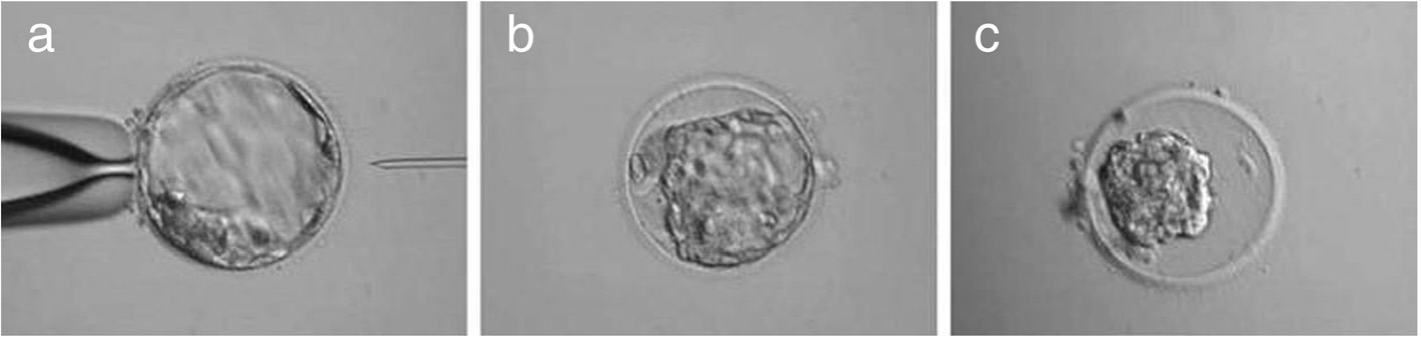
**Blastocoel reduction using a mechanical technique.** Artificial collapse of an expanded blastocyst is shown. (**a**,**b**) An ICSI needle is used to pierce the blastocyst in an area distal to the inner cell mass. (**c**) Within 5 minutes, blastocoelic fluid has leaked out and blastocoel volume is reduced. Magnification 300 ×.

Artificial shrinkage of fluid volume in blastocysts was performed to enhance survival. Expanding blastocysts with a blastocoel encompassing > ½ of the embryo were artificially collapsed prior to vitrification using an ICSI needle (Figure 2). The blastocyst was positioned on the holding pipette such that the inner cell mass was located at either the 12 or 6 o’clock position. An ICSI needle was pressed through the trophectoderm at the 3 o’clock position and advanced about mid way through the blastocyst. A slight negative pressure was maintained in the ICSI needle but blastocoelic fluid was not aspirated. The majority of blastocysts collapsed immediately upon piercing. The needle was then gently withdrawn. The embryo was incubated for five minutes to allow for additional shrinkage of the blastocoel before vitrification.

### Warming of embryos and assessment of survival

Cryoprotectant was removed by two sequential washes in warming solutions containing decreasing concentrations of sucrose (0.25 M and 0.125 M) in Global medium with 20% SPS. All steps were performed at 37°C with pre-equilibrated solutions. Warming solutions (0.5 ml) were placed in the center well of two organ culture dishes. A third wash dish was set up with culture medium for rinsing.

The Rapid-i straw was placed in a small dewar containing liquid nitrogen. The straw was cut and the inner Rapid-i stick with the embryos was firmly grasped, while still partially submerged in liquid nitrogen. The Rapid-i stick was then quickly pulled free of the straw and immersed in the first warming solution (0.25 M sucrose) and swirled to unload the embryos. The dish was then carefully examined under the dissecting scope and the embryos located. After two minutes, the embryos were moved to the second warming solution (0.125 M sucrose). After three minutes, the embryos were placed in the wash dish and incubated for an additional five minutes. Essentially the same procedure was used for warming embryos vitrified on the cryoloop. The embryos were unloaded by immersing the cryoloop directly in the first warming solution.

After the warming steps were completed, cleavage as well as blastocyst stage embryos underwent assisted hatching using a 1.48 μm diode laser. Two to three laser pulses were delivered to open the zona. The embryos were then transferred to a culture dish and examined under an inverted microscope at 400× magnification to assess for signs of cryoinjury and blastomere damage. Cleavage stage embryos were considered to have “survived” if at least half of the blastomeres were intact. Following warming, cleavage embryos were cultured for 48 hours to confirm continued developmental competency.

Cryosurvival of vitrified- warmed blastocysts, was determined on the basis of morphology immediately after warming. Blastocysts with dark, granular appearing cells and large areas of degeneration were identified as “non-surviving”. Warmed blastocysts were incubated for 2 hours and re-examined before transfer. Blastocoel expansion, trophectoderm organization and inner cell mass presence and cohesiveness were once again scored. A photographic record was kept of embryo morphology immediately after warming and just before transfer. After assessment of morphology, the embryos were moved to an organ culture dish containing Embryo Glue (VitroLife; Englewood, Colorado) in preparation for the transfer procedure.

### Embryo transfer and pregnancy assessment

Patients were prepared for frozen embryo transfer using endometrial priming with daily oral estradiol (6 mg) beginning on cycle day 1 and intramuscular (IM) progesterone (50–100 mg) starting on day 13. The endometrial lining was monitored for thickness and trilaminar appearance before starting progesterone therapy. Cycles were cancelled if the endometrial thickness was <7 mm. Transfer of vitrified-warmed blastocysts was performed after 6 days of progesterone treatment. Cleavage stage vitrified embryos were replaced after 5 days of progesterone. Embryo transfer was performed under transabdominal ultrasound guidance using a Wallace Sure-View catheter. Progesterone was continued for luteal support. Serum hCG levels were measured 15 days after transfer to detect pregnancy. A clinical pregnancy was confirmed by visualization of an intrauterine gestational sac with fetal heart activity on ultrasound 4 weeks after the embryo transfer. The implantation rate (IR) was calculated by dividing the number of gestational sacs by the number of embryos transferred. Ongoing pregnancy was defined as pregnancies continuing past 12 weeks gestation.

### Outcome parameters and data analysis

Outcome measures monitored were embryo survival (>50% intact), morphology at transfer, clinical pregnancy rate per transfer (CPR) and implantation rate. Clinical outcome data for frozen cycles between January 2011 and August 2012 were stratified according to carrier and cell stage. For data analysis, blastocyst FET cycles were further categorized according to the morphology of the transferred embryos post-warming. Embryos vitrified at the cleavage stage were monitored for resumption of mitosis, genomic activation and blastulation during the 48 hour culture interval before transfer. The student *t*-test, chi square test and Fischer’s exact test were used as appropriate to compare outcome parameters. Data is expressed as mean ± SD (standard deviation). P values of <0.05 were considered to be statistically significant. Statistical analysis was performed using the software Stats Direct (Cheshire, UK).

## Results

This study compared our initial results with the Rapid-i vitrification carrier to those achieved with our standard cryoloop method. Table 1 summarizes the results from vitrification-warming cycles with the Rapid-i (n = 95) and the cryoloop (n = 161). A total of 486 vitrified-warmed embryos were assessed and 92% of them were transferred. The data in the table is separated into four groups according to carrier and cell stage at vitrification. Patient ages in the four treatment groups were similar.

**Table 1:** Comparison of vitrification outcomes for day 3 cleavage and blastocyst stage embryos using different carriers

**Stage at Vitrification**	**Cleavage**		**Blastocyst**	
	**Rapid-i**	**Cryoloop**	**Rapid-i**	**Cryoloop**
**Patient age**	33.6 ± 3.3	34.1 ± 3.7	34.2 ± 3.8	34. ± 3.2
**No vitrification-warming cycles**	44	71	51	90
**No. vitrified embryos warmed**	92	139	92	163
**No. intact embryos on warming (%)**	91(99)	137 (99)	89 (97)	148 (91)
**Mean no. embryos thawed**	2.1 ± 0.7	2.0 ± 10.4	1.8 ± 0.7	1.8 ± 0.7
**No. transfers**	43	70	51	85
**No. embryos surviving and transferred**	85	130	88	143
**Mean no. embryos transferred**	2.0 ± 0.5	1.9 ± 0.4	1.7 ± 0.5	1.7 ± 0.5
**Implantation rate (%)**	31/85 (37)	46/130 (35)	43/88 (49)	54/143(38)
**Clinical pregnancy rate (%)**	20/43(47)	34/70 (49)	30/51 (59)	39/85 (46)
**Multiple pregnancy rate (%)**	2/20 (10)	10/34 (29)	8/30 (27)	9/39 (23)
**Miscarriage rate (%)**	2/43 (5)	3/70 (4)	5/51 (10)	6/85 (7)
**Deliveries**	15	29	16	26
**Infants**	16	36	18	36
**Boys/Girls**	8/8	17/19	9/9	15/21
**Ongoing pregnancies**	3	2	9	7

No significant differences between carriers for vitrification at either cell stage.

Data expressed as mean ± SD.

**Figure 3:**
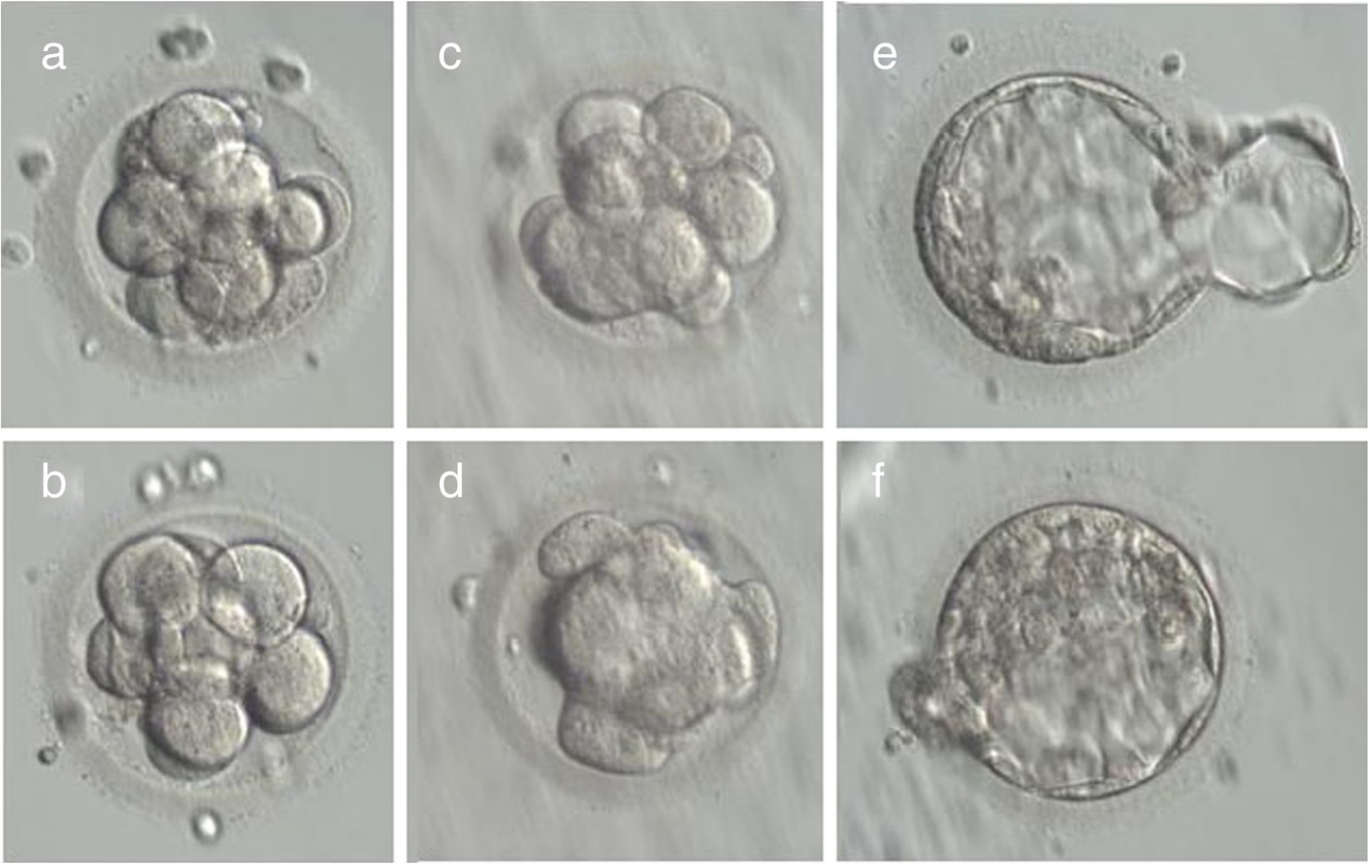
**Development of Rapid-i vitrified –warmed embryos.** Images of human embryos vitrified on Day 3 at the 8–10 cell stage using the Rapid-i carrier (**a,b**) Morphology immediately after warming, (**c,d**) Morphology after 24 hours of culture. Both embryos show signs of embryonic compaction and resumption of mitosis, (**e,f**) Embryos progressed to the blastocysts stage after 48 hours in culture. Transfer resulted in a twin pregnancy. Magnification 300 ×.

The clinical pregnancy and implantation rates with cleavage stage embryos vitrified on the Rapid-i (47% and 37%, respectively) were comparable to those obtained with the cryoloop (49% and 36%, respectively). The mean number of embryos warmed with Rapid-i and cryoloop was also similar (2.14 ± 0.71 and 2.01 ± 0.44, respectively). After warming, 99% of the embryos in both groups had 50% or more of their blastomeres intact. To date, we have had 15 deliveries of 16 healthy infants (8 boys and 8 girls) from Rapid-i vitrified cleavage stage embryos, with 3 pregnancies still on-going and 2 miscarriages. The percentage of pregnancies proceeding beyond 12 weeks gestation was 42% with the Rapid-i which was not significantly different from those with the cryoloop (44%). Resumption of mitosis after warming and the continued development of the 8–10 cell embryos during the 48 hour in vitro culture interval was prognostic of positive outcomes. In FET cycles where at last one of the cleavage stage embryos advanced to the expanded blastocyst stage, the CPR and IR in the Rapid-i treatment group were 67% (14/21) and 54% (22/41), respectively. In comparison, if embryos only reached the morula or early blastocyst stage, there was a marked decrease in both CPR (27%, 6/22 ) and IR (23%, 9/40), respectively (p < 0.05). A similar association between in vitro growth and outcome measures was observed with cryoloop vitrified day 3 embryos. Outcomes were significantly compromised when only early blastocysts/morula were available for transfer (CPR 33%, 15/45; IR 18%, 15/84) versus FET cycles with expanded/hatched blastocysts (CPR 76%, 19/25; IR 69%, 31/45; p < 0.05). Figure 3 illustrates typical morphology of vitrified 8-cell embryos 24 and 48 hours after warming.

Outcomes with blastocyst vitrification on the Rapid-i appeared somewhat higher than those achieved with cryoloop but the differences did not reach statistical significance. The clinical pregnancy rates were 59% vs. 46%, respectively; p = 0.199 and the implantation rates were 49% vs. 38 %; p = 0.128. The mean number of embryos warmed was 1.8 for both treatment groups. The survival rate of vitrified blastocysts was 97% with the Rapid-i versus 91% with the cryoloop (p = 0.07). Transfer of Rapid-i vitrified blastocysts has to date resulted in the birth of 18 healthy babies (9 boys and 9 girls), with 9 pregnancies still-ongoing and 5 miscarriages. Once again, the on-going pregnancy rate did not differ from that observed with cryoloop vitrification (49% vs. 39%).

**Figure 4:**
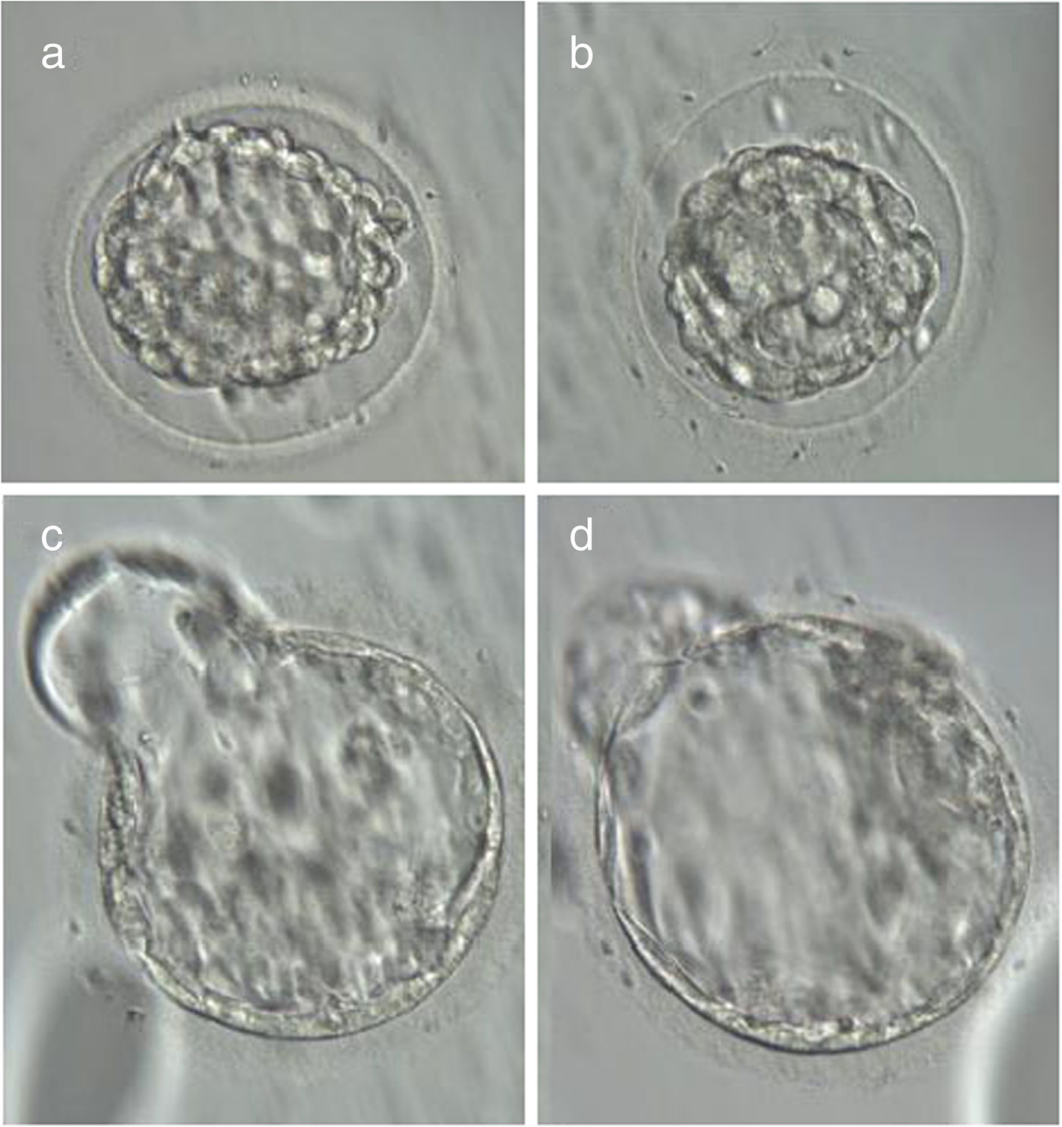
**Blastocyst stage vitrification and re-expansion after warming.** Human blastocysts vitrified with the Rapid-i. (**a,b**) Morphology immediately after warming, (**c,d**) Morphology two hours later. Both blastocysts now fully expanded and hatching out from the opening in the zona created after warming. Transfer resulted in a singleton pregnancy. Magnification 300 ×.

Blastocyst expansion and size of the blastocoel cavity were morphologic features closely linked to successful implantation of vitrified-warmed blastocysts. Blastocysts with high implantation potential appeared completely collapsed when recovered from the vitrification device and expanded by the time of transfer as shown in Figure 4.

**Figure 5:**
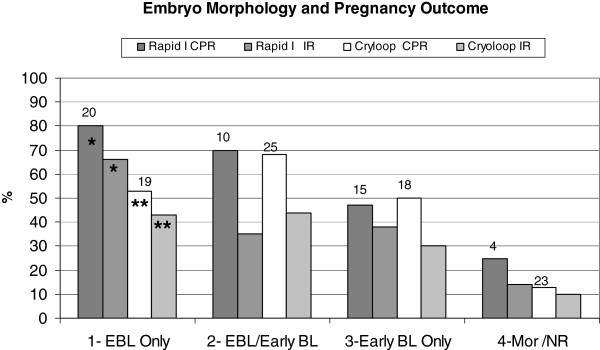
**Blastocyst morphology after warming and clinical outcomes.** Graph depicts relationship between embryo morphology and clinical outcomes in blastocyst (BL) vitrification-warming cycles. Embryos were graded as expanded blastocysts (EBL), early blastocysts, morula (Mor) or non re-expanded blastocysts (NR). Transfers were divided in to four general groups based on carrier used for vitrification and the morphology of the transferred embryos: 1-EBL only, 2-EBL/ Early BL (transfers with mixed stages), 3-Early BL only and 4-Mor/NR. Relationship between embryo morphology at transfer and resultant CPR and IR was examined. The number of transfers for each group and respective carrier is shown on the graph. Bars depict percent of transfers resulting in clinical pregnancy (CPR) and percent of embryos implanting (IR). * P < 0.05 CPR and IR for Rapid-i Group 1 vs. Groups 3 and 4 ** P < 0.05 CPR and IR for Cryoloop Group 1 vs. Group 4.

The clinical pregnancy rate and implantation data for varying qualities of embryos are presented in Figure 5. Transfers were grouped according to the morphology of the embryos 2 hours after warming. With the Rapid-i, 66% of expanded/hatched blastocysts (19/29) implanted and the CPR was 80% (16/20). In contrast, both the CPR and IR were significantly lower with early blastocysts, 47% (7/15) and 38% (10/26), respectively (p < 0.05). This difference between outcomes from advanced versus early blastocyts was not observed with cryoloop vitrified blastocysts. The clinical pregnancy rates were 53% (10/19) vs. 50% (9/18), respectively and implantation rates were 43% (13/30) vs. 30% (9/30), respectively. Regardless of vitrification carrier, the prognosis for patients with only morula or non re-expanded blastocysts for transfer was distinctly poorer with the implantation rates per embryo being 14% (1/7) for Rapid-i and 9.7% (3/31) for cryoloop.

## Discussion

The Rapid-i is a brand new vitrification carrier and this is one of the first clinical studies documenting its efficacy with human embryos at both early and late developmental stages. This is also the first report on live birth rates with the Rapid-i. The impact of the lower cooling rate during vitrification was of particular concern to us, especially as it relates to different embryonic stages and blastomere cell size. Our data however clearly indicate that the Rapid-i carrier can be effectively applied to both early cleavage as well as blastocyst stage embryos.

The basic premise underlying the design of new vitrificaton systems and carriers has, to date, been focused on achieving high cooling rates. As pointed out by Seki and Mazur [34], cryoinjury may in fact be more related to recrystallization during warming rather than the failure to vitrify. In that study, the authors examined the relationship between cooling versus warming rates and vitrification results with mouse oocytes. They concluded that a warming rate of at least 3000°C/min was necessary to achieve 80% survival. Moreover, their data indicated that if the warming was quick enough, a cooling rate of as low as −200°C/min should be adequate for successful vitrification.

Our present data with human embryos certainly concurs with the relative importance of the warming over the cooling rate. The warming temperature of 7700°C/min with the Rapid-i was clearly sufficient to give outcomes comparable to an open system like the cryoloop, despite a 15-fold lower cooling rate of −1220°C/min. The 59% CPR and 49% implantation rate achieved with Rapid-i blastocyst vitrification closely parallels pregnancy outcomes from fresh single embryo transfer of blastocysts at our IVF center (unpublished results). We also found that the lower cooling rate with the Rapid-i was not a deterrent to successful vitrification of day 3 cleavage embryos despite the larger size of the individual blastomeres. According to the SART (Society for Assisted Reproductive Technology) registry’s 2011 national summary data, young patients under 35 having a fresh transfer had a 36% implantation rate. It was reassuring that we were able to achieve this same implantation rate with Rapid-i vitrified 8-cell embryos.

Concerns have been raised regarding the safety of open vitrification carriers for the cryopreservation of reproductive cells [2,20-22,35]. Although, there have been no reported incidents of human reproductive tissue contamination during storage in liquid nitrogen tanks to date, there has been a movement toward the use of “closed” sealed systems for human gamete/embryo cryopreservation. The long-standing argument for use of open systems has been that the benefit of higher cooling rates and ease of embryo loading/recovery far outweigh the theoretical risks of using such and open system. This former objection is rapidly becoming obsolete with accumulating pregnancy outcome data from closed vitrification carriers [6]. The current data set provides further evidence to suggest that a closed vitrification carrier can be used effectively for embryos at both early and late stages. Despite the far lower cooling and warming rates with the Rapid-i (−1220°C/min, +7700°C/min) compared to the HSV straw (−2900°C/min, +25,000°C/min) [26,27], clinical outcomes more than matched those with the HSV (CPR 45%, IR 31%) [23]. Transitioning to the Rapid-i closed carrier was aided by its clever design. Its resemblance to the cryoloop as far as embryo loading and recovery made it technically easy to use.

It should be noted that with cryopreservation at the blastocyst stage, it was important to critically assess the degree of expansion and alter the vitrification protocol accordingly. Blastocyst collapse was integral to successful vitrification of expanded blastocysts. Cell death and DNA damage is lower if the size of the blastocoelic cavity is reduced prior to vitrification [36]. Vanderzwalmen and colleagues first described the benefit of blastocoel shrinkage to improve cryosurvival [18]. A variety of methods have been applied to reduce blastocoelic volume prior to vitrification leading to improvements in post-warming survival and ultimately, clinical outcomes [14,15,18,37,38]. Mukaida and colleagues reported an implantation rate of 47% with mechanically or laser collapsed blastocysts vitrified on cryoloops [37]. We had a similarly high 49% implantation rate with mechanically collapsed blastocysts cryopreserved using the the Rapid-i closed vitrification system. In contrast, Hashimoto et al. vitrified blastocysts on the Rapid-i without collapsing and reported clinical outcomes similar to our own [29]. It is likely that the specific vitrification protocol in combination with the type of carrier and the morphology of the blastocyst being cryopreserved may dictate whether or not collapsing is necessary.

## Conclusions

In summary, the Rapid-i offers an excellent alternative to existing open vitrification devices for embryo cryopreservation at the 8–10 cell stage as well as at the blastocyst stage. Use of this type of “closed” system that prevents direct contact between the embryos and liquid nitrogen reduces the potential risk of sample cross-contamination or infection. These preliminary data and live birth outcomes have paved the way toward transitioning to a closed vitrification system in our own IVF program. The ease of embryo loading and unloading played an important role in the selection and acceptance of this particular device as opposed to other commercially available closed vitrification devices. Additional data from other centers are needed to further corroborate these findings.

## Competing interests

The authors declare that they have no competing interests.

## Authors’ contributions

ND designed the study, performed procedures, collected and analyzed the data and drafted the manuscript. JMB, CA and TF critically reviewed the data and helped draft the manuscript. All authors read and approved the final manuscript.
